# USP Reference Standard Monoclonal Antibodies: Tools to Verify Glycan Structure

**DOI:** 10.3390/ph15030315

**Published:** 2022-03-05

**Authors:** Jingzhong Guo, Huiping Tu, Li Jing, Diane McCarthy, Fouad Atouf

**Affiliations:** Global Biologics, Science Division, United States Pharmacopeia, Rockville, MD 20852, USA; tim.guo@usp.org (J.G.); hpt@usp.org (H.T.); li.jing@usp.org (L.J.); diane.mccarthy@usp.org (D.M.)

**Keywords:** glycan analysis, monoclonal antibody, NISTmAb, USP

## Abstract

The glycan profile is a critical quality attribute for pharmaceutical monoclonal antibodies due to the potential physiological impact of the glycan composition when used as a drug product. Monoclonal antibody reference standards are useful as system suitability samples for glycan profile testing. The development of future glycan profiling techniques could be better evaluated by testing well-characterized reference standards. The USP has introduced monoclonal antibody reference standards (i.e., USP mAb 001 RS, USP mAb 002 RS, and USP mAb 003 RS) with the glycan profiles reported herein that can be used to assess the analytical testing of monoclonal antibody glycan profiles. Comparison of the USP reference standards to other available reference standards (NISTmAb) is presented. The glycan profile of the USP monoclonal antibody reference standards covers a range of glycan species that complements other available reference standards. The USP mAb reference standards are a valuable tool that can be used to verify the glycan structure and provide the system suitability of analytical methods.

## 1. Introduction

Monoclonal antibodies have become a valuable biologic that the clinician may use to treat a myriad of illnesses. It is the specificity of binding and mode of action of the monoclonal antibody toward its target that allows these therapeutics to precisely treat the illness. There are numerous structural features that contribute to the specific actions of a given monoclonal antibody, including the glycan structure associated with the monoclonal antibody [1,2,3,4,5,6,7,8,9]. The glycan structure of a monoclonal antibody is typically heterogeneous with a predominance of a particular structure(s) that can contribute to stability, bioactivity, pharmacokinetics and pharmacodynamics (PK/PD), and immunogenicity of the molecule [4,5,6,9]. For these reasons, the glycan structure of a monoclonal antibody product is a critical quality attribute that must be evaluated through analytical characterization during the development and commercialization of a monoclonal antibody product.

Analytical characterization of the glycan structure of a monoclonal antibody requires scientifically sound analytical procedures that are accurate, precise, and reproducible [1,2,3,7,8]. To demonstrate that an analytical procedure is scientifically sound often requires characterizing a reference standard in parallel with a test sample to demonstrate that the analytical procedure was performing properly as expected. For the determination of glycan structure, this requires a reference standard that has been highly characterized, using one or more orthogonal methods, to fully understand the glycan structure of the molecules in the reference standard.

A monoclonal antibody reference standard, with extensive glycan characterization [1], is available from the National Institute of Standards and Technology designated as NIST Reference Material 8671, NISTmAb. The United States Pharmacopeia (USP) developed three monoclonal antibody reference standards (i.e., USP mAb 001 RS, USP mAb 002 RS, and USP mAb 003 RS) that may be used as control materials to demonstrate whether glycan characterization procedures provide an accurate result. The USP mAb reference standards are different proteins of the same IgG1 subclass yet provide sufficient variability to examine a broad spectrum of glycan structures. Glycan characterization is commonly performed by analysis of glycans released from the protein backbone. As a result of lacking UV absorption chromophores, the release glycans are usually derivatized by introducing fluorescence tags such as 2-aminobenzamide (2-AB) and 2-aminoanthranilic acid (2-AA) to facilitate High-Performance Liquid Chromatography—Fluorescent Detection (HPLC-FLR) [10]. HPLC-FLR is suitable for profiling and quantitative analysis but is limited for structure identification [10,11]. In recent years, Mass Spectrometry (MS) has been increasingly utilized with FLR for glycan identification and providing structure information. In this study, we used a rapid labeling kit-RapidFluor-MS (RFMS) which provides good sensitivity for FLR and MS detections of N-glycans [3,10,11]. The glycan structures of these three USP mAb reference standards are provided in this report to further their use in supporting glycan structure characterization studies.

## 2. Results and Discussion

### 2.1. Glycan Profiling and Proposed Identifications

The USP reference standard antibodies glycan profiles were determined using methods previously described [2,3]. The highest normalized glycan abundance in all three antibodies was F(6)A2 at 44.46% (mAb 001, Table 1), 67.81% (mAb 002, Table 2), and 49.56% (mAb 003, Table 3). A normalized abundance cutoff was set to 0.1%. Minor glycans with normalized abundance below 0.1% were not included in this article. This does not mean that minor glycans are not important. Some minor glycans may have even stronger bioactivities and be responsible for immune responses. This report is to present the major glycans readily detected in the three USP reference standard antibodies. The identification of very minor glycans in these reference standards is beyond the scope of this study. We would encourage the readers to identify vary minor glycans if more sensitive instrumentation or methodologies are accessible. The number of structural elements within the glycan profile of each monoclonal antibody is summarized in Table 4. The co-elution of glycans and unknown glycans, glycans that were not included in the database library, were observed for all three antibodies as shown in Table 1, Table 2 and Table 3. Each of the co-eluting species was mass-confirmed through manual inspection of the mass spectrometry data. The normalized abundance is expressed as the total area of the FLR peak and includes the contribution of co-eluting species in Table 1, Table 2 and Table 3, if they are present. In some cases, more than one glycan isomers were proposed under the same FLR peak. These isomers may coexist, or it is possible that only one of them dominates, as they cannot be distinguished with the current approach. This indicates the limitation of the current approach, although it is widely used currently in industry [3,8]. A combination of this approach with automatic analysis of LC-MSMS data and ion mobility data may help to differentiate the glycan isomers precisely [9].

**Figure 1:**
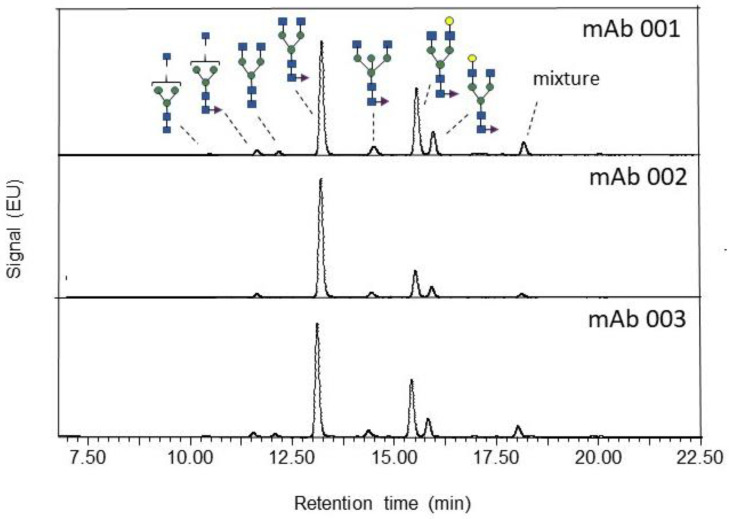
Annotated FLR trace depicting the N-linked glycans derived from USP mAb 001, mAb 002, and mAb 003. Peaks for glycans A1, F6A1, A2, F6A2, F6A2B, F(6)A2[6]G(4)1, and F(6)A2[3]G(4)1 are identified. A peak representing a mixture of F(6)A2[6]G(4)1Ga1, F(6)A2G(4)2 = G2F, F(6)A2[3]G1Ga1, and F(6)A2[3]G(4)1S(3)1 was observed.

**Figure 2:**
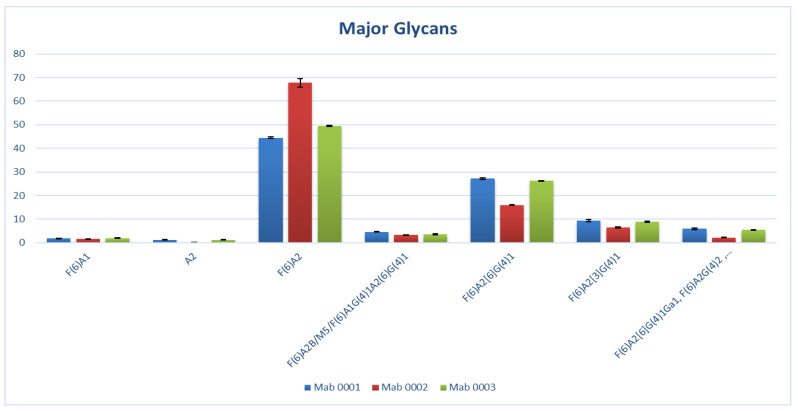
Abundance level of various glycans found in USP mAb 001, mAb 002, and mAb 003. The error bars indicate three standard deviations of the averaged abundance from four injections.

The chromatogram observed with USP mAb 001, USP mAb 002, and USP mAb 003 (Figure 1) resembles that of other mAbs [3]. A comparison of the major glycans observed among these USP monoclonal antibody reference standards is shown in Figure 2. The low standard deviation of glycan abundance in Figure 2 indicates a good reproducibility of the assay. Most of the characteristic structural elements observed in the glycan profile of mAbs are summarized for the three USP monoclonal antibody reference standards in Table 4.

### 2.2. Applications for Verification Glycan Structures and Assessing Critical Quality Attributes of mAbs

Glycans associated with protein products are a diverse group of molecules that are challenging to characterize. *N*-glycosylation of the Fc region of an IgG molecule has been identified as a critical quality attribute for some monoclonal antibodies due to the recognized effector functions (e.g., ADCC, CDC, and PK) of certain glycan species [4,5]. Monoclonal antibody *N*-linked glycans with terminal sialic acid can result in antibodies with longer half-lives in the body than non-sialylated antibodies, monoclonal antibodies with a high content of glycans with terminal mannose are cleared more quickly from the blood, and the absence of core fucose on monoclonal antibody glycans can enhance the affinity for the Fc region with the FcγRIIIa receptor, increasing the ADCC [6]. Each of the glycan elements including sialylated, and many more, can be found in the USP mAb reference standards.

The five most abundant glycans that have been reported for USP mAb 001, mAb 002, mAb 003, and NIST mAb are shown in Table 5. The results indicate that the USP monoclonal antibody reference standards and the NISTmAb are similar with regard to glycan profiles, with the notable exception of USP mAb 002. USP mAb 002 shows a higher level of F(6)A2 and lower levels of F(6)A2[6]G(4)1, F(6)A2[3]G(4)1, and F(6)A2G(4)2 when compared to other glycan reference standards (Table 5). These differences observed with USP mAb 002 make it valuable as a unique sample when evaluating the system suitability of analytical techniques to determine monoclonal antibody glycan profiles. Comparison of the glycan profiles beyond the five most abundant glycans as shown in Table 5 becomes difficult given that some of the identified glycans that are less abundant co-elute. A further complication with the quantitative comparison of low abundance glycans is possible with 47, 45, and 40 glycans observed with USP mAb 001, USP mAb 002, and USP mAb 003, respectively. Fifty-seven glycans were reported for NIST mAb [1], and in another study, 35 glycans were reported for NIST mAb [3]. This variation may have an impact on the comparison of relative glycan abundance determination between reference standards based on the sum of the total peak area. The low abundance glycans are useful when evaluating if a particular analytical method can detect the entire glycan profile.

There is value in having multiple well-characterized monoclonal antibodies that can serve as reference standards to verify the various analytical glycan profiling techniques now and in the future. Although quantitative comparison of glycan profiles among the monoclonal antibody reference standards may be difficult for less abundant glycans (i.e., those glycans less than the five most abundant), their use in the evaluation of the analytical methods with regard to resolution and sensitivity is significant. Discrepancies observed with multiple glycosylation analytical methods and multiple laboratories highlight the value in having available multiple reference standards to use for analytical method verification and control [7].

As previously reported [8,9], without validated reference standards, manufacturers cannot create an in-depth glycan profile that includes the minor glycans that may be present or validate the suitability of their equipment for such an analysis. The major glycans present in the USP monoclonal antibodies cover a quantitative range (Table 5) which gives added benefit in their use in evaluating the suitability of an analytical method. Therein lies the value of the USP mAb 001, mAb 002, and mAb 003 reference standards available from the United States Pharmacopeia.

## 3. Materials and Methods

### 3.1. Reagents and Materials

The mAb 001, mAb 002, and mAb 003 reference standards were obtained from the United States Pharmacopeia. RFMS-labeled human IgG (RFMS Glycan Performance Test Standard, product number 186007983) and mouse IgG (Intact mAb Mass Check Standard, product number 186006552) were purchased from Waters Corp (Milford, CT, USA). Ammonium formate solution, Glycoworks^®^ RapiFluor-MS Performance Test Standard, Glycoworks Rapi-Fluor-MS dextran calibration ladder, and a Glycoworks Rapi-Fluor-MS 24-sample *N*-Glycan Kit were obtained from Waters Corp. The complete kit consists of three modules: deglycosylation, labeling, and clean-up. When combined, the kits contain all of the supplies needed to complete the sample preparation, and all three were utilized in this project. The Glycoworks Deglycosylation Module contained Intact mAb Mass Check Standard, Glycoworks Rapid PNGase F and Buffer, and RapiGestTM SF. The Glycoworks^®^ RapiFluor-MSTM Labeling Module contained the Glycoworks^®^ RapiFluor-MSTM Reagent and Glycoworks^®^ Reagent Solvent Anhydrous Dimethylformamide (DMF). The Glycoworks^®^ RapiFluor-MSTM Clean-up Module contained a Glycoworks^®^ HILIC μ-Elution Plate, Glycoworks^®^ SPE elution buffer (200 mM ammonium acetate, 5% acetonitrile (ACN)), and Glycoworks^®^ Sample Diluent (DMF). The Glycoworks^®^ RapiFluor-MSTM Sample Collection Module contained sample collection tubes, a waste tray, and a collection tray. The Amicon spin concentrator (0.5 mL, 10 K), LC-MS grade water, and acetonitrile were purchased from Thermo Fisher Scientific.

### 3.2. PNGase Digestion to Remove Glycans from mAbs

*N*-linked glycans derived from the mAb 001, mAb 002, and mAb 003 were prepared in duplicate. The samples were diluted and buffer-exchanged to a concentration of 2 mg/mL in a phosphate-buffered saline solution (100 mM sodium phosphate, 150 mM sodium chloride, pH 7.2). In brief, in an Amicon spin concentrator, add 10 µL of 10 mg/mL sample and 40 µL PBS solution, and then centrifuge at 7500× *g* at 8 °C for 15 min. After washing 3 times with 100 µL of PBS and centrifugation, collect the sample and bring the volume to 50 µL. In a 1 mL Eppendorf tube, add 7.5 µL (15 µg) buffer-exchanged sample, 5.3 µL of water, and 6 µL of a 5% RapiGest solution. The proteins were denatured at 95 °C for 3 min and then cooled to the ambient temperature. Next, add 1.2 µL of Rapid PNGase F, aspirate to mix well, and then incubate at 55 °C for 5 min to release the *N*-linked glycans as their glycosylamines.

### 3.3. RFMS Labeling of Released Glycans

Following the digestion, the amino group of the released glycosylamines was labeled with RFMS. A 24-reaction kit was used, and the RFMS reagent (9 mg) was dissolved in 131 μL of anhydrous DMF. A 12 μL aliquot of this solution was added to each glycan sample and allowed to react at the ambient temperature for 5 min. Then, the reaction was quenched with the addition of a 358 μL aliquot of ACN, which also adjusted the solution to an appropriate organic solvent concentration for Hydrophilic Interaction Liquid Chromatography (HILIC)-based purification.

### 3.4. Glycan Purification

The derivatized *N*-linked glycans were purified using a HILIC μ-Elution plate. The medium was first washed with three 200 μL aliquots of water, which was followed by three 200 μL of 85% ACN in water. Then, the samples (400 µL) were loaded onto the medium and washed with two 600 μL of washing solution containing 90% ACN and 1% water in water. The glycans were eluted by three 30 μL aliquots of an SPE Elution Buffer (200 mM ammonium acetate in 5% ACN). The eluate was diluted with 310 µL of GlycoWorks SPE Diluent and transferred into autosampler vials for Liquid Chromatography Mass Spectrometry (LC-MS) analysis.

### 3.5. UPLC-MS Analysis

A Waters ACQUITY H-class Ultra-Performance Liquid Chromatography (UPLC) system, consisting of a quaternary solvent manager, a sample manager, set to 5 °C, a column manager, operating at 60 °C, and a fluorescence (FLR) detector (excitation wavelength 265 nm, emission wavelength 425 nm, data collection rate of 2 Hz) was used for the separations. The glycans were separated with a Waters ACQUITY UPLC Glycan BEH Amide column (2.1 × 150 mm, 1.7 μm particle size, 130 Å pore size). Mobile Phase A was a 50 mM ammonium formate solution (pH 4.4), and Mobile Phase B was neat ACN. Analyte separation was accomplished by gradient elution using a gradient running from 75 to 54% Mobile Phase B over 35 min at a flow rate of 0.4 mL/min. For the mAb 001, mAb 002, and mAb 003 analysis, the UPLC system was coupled to a Waters Synapt-G2S QT of MS and was operated in its positive sensitivity mode to monitor the *m*/*z* range from 600 to 2500 at a scan rate of 2 Hz. The capillary voltage was set to 3 kV, and a cone voltage of 40 V was used. The source temperature was 120 °C, and the desolvation temperature was set at 350 °C. For all Mass Spectrometry (MS) analysis, a 100 pmol/μL solution of [Glu1]-fibrinopeptide B in 50%/50%/0.1% water/ACN/formic acid was used for Lockspray calibration, and the 2+ ion at *m*/*z* 785.8427 was used for calibration. Along with sample injections, RFMS-labeled dextran was injected at the beginning and end of the sequence as standards for determining GU values (Glucose Units) of all detected glycans. The Glycoworks^®^ RapiFluor-MS Performance Test Standard was also injected prior to the samples and used for system suitability tests. Duplicate injections were performed for each of the duplicate sample preparations.

### 3.6. Data Analysis

HILIC-UPLC/FLR/MS data were processed and analyzed using the Glycan Assay (FLR with MS confirmation) workflow within UNIFI. This workflow first converted the retention times of the labeled glycans samples to Glucose Units (GU) based on a cubic spline calibration curve against a dextran ladder labeled with RFMS. Then, these GU values were searched against the RFMS Glycan GU Scientific Library housed within UNIFI for glycan structural identification. The library searches used a GU tolerance of 0.3 GU and a mass error of 10 ppm. For relative quantitation, the FLR peak area for each glycan was expressed as a percentage of the total summed peak area for all the glycans identified. In the case of co-eluting glycans, mass-confirmed through manual inspection of the data, the total peak area and percentage amounts are reported. The relative abundance of glycans is from an average of four injections of the duplicate sample preparations.

## 4. Conclusions

In this study, we have presented the glycan profiles and proposed identifications of all major and most minor glycans released from the three recently released monoclonal antibody reference standards (i.e., USP mAb 001 RS, USP mAb 002 RS, and USP mAb 003 RS). The glycan profile of the USP monoclonal antibody reference standards covers a range of glycan species that complements other available reference standards. The USP mAb reference standards can be used as a valuable tool to verify glycan structures and provide the system suitability of analytical methods for glycan profile testing of pharmaceutical mAb products.

## Figures and Tables

**Table 1:** A list of the mass-confirmed N-linked glycans derived from the USP mAb 001. The table also includes the observed retention times, normalized peak area percentage, expected and observed GU values, expected and observed masses and *m*/*z* values, and the mass error expressed in parts per million (ppm). In the case of co-eluting glycans, total peak area percentages are reported. In case of glycan isomers proposed under the same glycan peak, the isomers may coexist or one of them dominates, as they cannot be distinguished with the current approach.

Component Name	Observed RT (min)	Peak Area (%)	Expected Glucose Units	Glucose Units	Expected Mass (Da)	Observed Mass (Da)	Observed *m*/*z*	Mass Error (ppm)
F(6)M3	9.92	0.	4.77	4.79	1367.5603	1367.5689	684.7923	6.31
A1	10.52	0.3	4.96	4.99	1424.5818	1424.5943	713.3049	8.83
F(6)A1	11.72	2.0	5.31	5.36	1570.6397	1570.6523	786.3340	8.04
A2	12.15	1.2	5.49	5.53	1627.6611	1627.6721	814.8439	6.76
F(6)A2	13.31	44.5	5.82	5.86	1773.7190	1773.7381	887.8769	8.82
M5 Isomer	14.30	0.1	6.19	6.18	1545.6080	1545.6171	773.8164	5.91
F(6)A2B	14.60	4.7	6.19	6.27	1976.7984	1976.8105	989.4131	6.14
M5			6.19		1545.6080	1545.6167	773.8162	5.65
F(6)A1G(4)1			6.22		1732.6925	1732.7011	867.3584	4.99
A2[6]G(4)1			6.26		1789.7139	1789.7197	895.8677	3.23
A2[3]G(4)1	15.08	0.2	6.38	6.43	1789.7139	1789.7269	895.8713	7.25
F(6)A2[6]G(4)1	15.67	27.2	6.53	6.62	1935.7719	1935.7857	968.9007	7.17
F(6)A2[3]G(4)1	16.08	9.36	6.69	6.76	1935.7719	1935.7847	968.9002	6.65
F(6)M5A1	16.44	0.1	6.91	6.87	1894.7453	1894.7571	948.3864	6.41
F(6)M4A1G(4)1	17.10	0.4	7.12	7.10	1894.7453	1894.7551	948.3854	5.35
F(6)A1G(4)1Ga(3)1			7.12					
M6			7.11		1707.6609	1707.6687	854.8422	4.61
M6D3			7.12					
Unknown 1	17.25	0.8		7.15		2023.7993	1012.907	
M6D1	17.35	0.3	7.14	7.18	1707.6609	1707.6701	854.8429	5.43
A2G(4)1Ga(3)1			7.05		1951.7668	1951.7815	976.8986	7.55
A2G(4)2			7.10					
F(6)A3G(4)1	17.72	0.2	6.91	7.29	2138.8512	2138.8661	1070.4410	7.08
F(6)A2[3]BG(4)1			6.97					
F(6)A3G(4)1 iso			7.02					
F(6)A3G(4)3S(3,3,3)3	17.80	0.3	7.41	7.34	2226.8673	2226.8789	1114.4770	5.24
F(6)A2[6]G(4)1Ga(3)1	18.31	5.9	7.38	7.51	2097.8247	2097.8369	1049.9263	5.84
F(6)A2G(4)2			7.43					
F(6)A2[3]G(4)1Ga(3)1			7.55					
F(6)A2[3]G(4)1S(3)1			7.53		2226.8673	2226.8837	1114.4497	7.39
M4A1G(4)1Ga(3)1	18.64	0.2	7.42	7.63	1910.7402	1910.7541	956.3849	7.28
M5A1G(4)1			7.43					
F(6)M5A1G(4)1	19.04	0.2	7.74	7.77	2056.7981	2056.8119	1029.4138	6.71
F(6)M4A1G(4)1Ga(3)1	19.56	0.2	7.80	7.93	2056.7981	2056.8123	1029.4140	6.90
M7D3			7.81		1869.7137	1869.7283	935.8720	7.84
M7	20.03	0.1	8.0	8.14	1869.7137	1869.7269	935.8713	7.09
M7D1			8.03					
F(6)A3G(4)1Ga(3)1 iso			7.88		2300.9041	2300.9221	1151.4689	7.88
F(6)A2G(4)2S(3)1	20.16	0.5	8.12	8.19	2388.9201	2388.9355	1195.4756	6.46
F(6)A2G(4)2S(6)1	20.37	0.3	8.55	8.26	2388.9201	2388.9361	1195.4759	6.71
F(6)A2G(4)2Ga(3)1	20.72	0.1	8.25	8.39	2259.8775	2259.8925	1130.9541	6.65
F(6)M4A1G(4)1Sg(6)1			8.58		2201.8356	2201.8505	1101.9331	6.79
F(6)A2G(4)2Ga(3)1 iso			8.30		2259.8775	2259.8925	1130.9541	6.65
M8	22.03	0.3	8.84	8.90	2031.7665	2031.7833	1016.8995	8.28
M8D1D3			8.82					
M8D2D3			8.76					
F(6)A2G(4)2S(3,3)2	22.25	0.3	8.85	8.99	2680.0155	2680.0299	1341.0228	5.38

Nomenclature: F: fucose; G: galactose; Sg: N-glycolylneuraminic acid; Sx, number (x) of sialic acids linked to galactose; Ga: α1,3-linked galactose; A1: monoantennary; A2: biantennary; Mx: number (x) of mannose on core GlcNAcs; D1 indicates that the α1,2 mannose is on the Manα1,6Manα1,6 arm, D2 indicates that the α1,2 mannose is on the Manα1,3Manα1,6 arm, D3 indicates that the α1,2 mannose is on the Manα1,3 arm of M6 and on the Manα1,2Manα1,3 arm of M7 and M8; B: bisecting GlcNAc linked_1,4 to 1,3 mannose; Numbers with parentheses indicate the preceding monosaccharide’s linkage and those in brackets define to which core mannose is extended, if it needed to be defined. Numbers not in parentheses indicate the amount of the preceding feature. For example, F(6)A2[3]G(4)1Ga(3)1 represents a core fucosylated (α1,6-linked) bianntennary glycan with a β1,4-linked galactose directly attached to the α1,3-linked core mannose and an α1,3-linked galactose attached to the β1,4-linked galactose.

**Table 2:** A list of the mass-confirmed N-linked glycans derived from the USP mAb 002. The table also includes the observed retention times, normalized peak area percentage, expected and observed GU values, expected and observed masses and *m*/*z* values, and the mass error expressed in parts per million (ppm). In the case of co-eluting glycans, total peak area percentages are reported. In case of glycan isomers proposed under the same glycan peak, the isomers may coexist or one of them dominates, as they cannot be distinguished with the current approach.

Component Name	Observed RT (min)	Peak Area (%)	Expected Glucose Units	Glucose Units	Expected Mass (Da)	Observed Mass (Da)	Observed *m*/*z*	Mass Error (ppm)
M3	8.74	0.1	4.36	4.41	1221.5024	1221.5113	611.7635	7.31
A1	10.52	0.1	4.96	4.99	1424.5818	1424.5911	713.3034	6.55
F(6)A1	11.7	1.7	5.31	5.36	1570.6397	1570.6483	786.3320	5.50
A2	12.24	0.2	5.49	5.53	1627.6611	1627.6797	814.8427	5.30
F(6)A2	13.28	67.8	5.82	5.85	1773.7190	1773.7337	887.8747	8.30
M5 Isomer	14.25	0.1	6.19	6.16	1545.6080	1545.6187	773.8172	6.94
F(6)A2B	14.52	3.3	6.19	6.25	1976.7684	1976.8115	989.4136	6.64
M5			6.19		1545.6080	1545.6217	773.8187	8.88
F(6)A1G(4)1			6.22		1732.6925	1732.7069	867.3613	8.33
A2[6]G(4)1			6.26		1789.7139	1789.7231	895.8694	5.16
F(6)A3	15.32	0.1	6.27	6.53	1976.7984	1976.8125	989.4141	7.15
F(6)A2[6]G(4)1	15.60	16.0	6.53	6.60	1935.7719	1935.7871	968.9014	7.87
F(6)A2[3]G(4)1	15.99	6.5	6.69	6.73	1935.7719	1935.7845	968.9001	6.52
F(6)M5A1	16.34	0.1	6.89	6.87	1894.7453	1894.7569	948.3863	6.14
F(6)M4A1G(4)1	17.00	0.1	6.99	7.05	1894.7453	1894.7607	948.3882	8.14
F(6)A1G(4)1Ga(3)1			7.02					
M6			7.11		1707.6609	1707.6737	854.8447	7.46
M6D3			7.12					
Unknown 1	17.04	0.1		7.08		2023.8045	1012.9101	
M6D1	17.27	0.3	7.14	7.15	1707.6609	1707.6703	854.8430	5.47
A2G(4)1Ga(3)1			7.05		1951.7668	1951.7771	976.8964	5.20
A2G(4)2			7.10					
F(6)A3G(4)1	17.6	0.1	6.91	7.27	2138.8512	2138.8667	1070.4412	7.36
F(6)A2[3]BG(4)1			6.97					
F(6)A3G(4)1 iso			7.02					
F(6)A3G(4)3S(3,3,3)3	17.59	0.2	7.41	7.26	2226.8673	2226.8667	1114.4503	8.06
F(6)A2[3]G(4)1S(3)1			7.53					
F(6)A2[6]G(4)1Ga(3)1	18.20	2.2	7.38	7.48	2097.8247	2097.8395	1049.9276	6.93
F(6)A2G(4)2			7.43					
F(6)A2[3]G1(4)Ga(3)1			7.55					
F(6)A2[3]G(4)1S(3)1			7.53		2226.8673	2226.8859	1114.4508	8.51
M4A1G(4)1Ga(3)1	18.51	0.1	7.42	7.58	1910.7402	1910.7521	956.3873	6.35
M5A1G(4)1			7.43					
F(6)M5A1G(4)1	18.92	0.1	7.74	7.73	2056.7981	2056.8119	1029.4138	6.78
F(6)M4A1G(4)1Ga(3)1	19.46	0.1	7.80	7.93	2056.7981	2056.8099	1029.4128	5.81
M7D3			7.81		1869.7137	1869.7253	935.8705	6.07
M7	19.89	0.1	8.0	8.08	1869.7137	1869.7283	835.8720	7.67
M7D1			8.03					
F(6)A2G(4)2S(3)1	20.05	0.1	8.12	8.14	2388.9201	2388.9409	1195.4783	8.77
F(6)A2G(4)2S(6)1	20.25	0.1	8.55	8.22	2388.9201	2388.9361	1195.4759	6.76
M8	21.9	0.1	8.84	8.85	2031.7665	2031.7805	1016.8995	7.16
M8D1D3			8.82					
M8D2D3			8.76					
F(6)A3G(4)3 iso			8.80		2462.9569	2462.9725	1232.4941	6.35
F(6)A2G(4)2S(3,3)2	22.23	0.1	8.85	8.98	2680.0155	2680.0311	1341.0234	5.38

Nomenclature: F: fucose; G: galactose; Sg: N-glycolylneuraminic acid; Sx, number (x) of sialic acids linked to galactose; Ga: α1,3-linked galactose; A1: monoantennary; A2: biantennary; Mx: number (x) of mannose on core GlcNAcs; D1 indicates that the α1,2 mannose is on the Manα1,6Manα1,6 arm, D2 indicates that the α1,2 mannose is on the Manα1,3Manα1,6 arm, D3 indicates that the α1,2 mannose is on the Manα1,3 arm of M6 and on the Manα1,2Manα1,3 arm of M7 and M8; B: bisecting GlcNAc linked_1,4 to 1,3 mannose; Numbers with parentheses indicate the preceding monosaccharide’s linkage and those in brackets define to which core mannose is extended, if it needed to be defined. Numbers not in parentheses indicate the amount of the preceding feature. For example, F(6)A2[3]G(4)1Ga(3)1 represents a core fucosylated (α1,6-linked) bianntennary glycan with a β1,4-linked galactose directly attached to the α1,3-linked core mannose, and an α1,3-linked galactose attached to the β1,4-linked galactose.

**Table 3:** A list of the mass-confirmed *N*-linked glycans derived from the USP mAb 003. The table also includes the observed retention times, normalized peak area percentage, expected and observed GU values, expected and observed masses and *m*/*z* values, and the mass error expressed in parts per mil lion (ppm). In the case of co-eluting glycans, total peak area percentages are reported. In case of glycan isomers proposed under the same glycan peak, the isomers may coexist or one of them dominates, as they cannot be distinguished with the current approach.

Component Name	Observed RT (min)	Peak Area (%)	Expected Glucose Units	Glucose Units	Expected Mass (Da)	Observed Mass (Da)	Observed *m*/*z*	Mass Error (ppm)
A1	10.46	0.5	4.96	4.97	1424.5818	1424.5891	713.3024	5.01
F(6)A1	11.62	2.0	5.31	5.33	1570.6397	1570.6483	786.3320	5.49
A2	12.15	1.3	5.49	5.50	1627.6611	1627.6713	814.8435	6.29
F(6)A2	13.19	49.6	5.82	5.83	1773.7190	1773.7303	887.8730	6.39
M5 Isomer	14.25	0.1	6.19	6.13	1545.6080	1545.6153	773.8155	4.74
F(6)A2B	14.52	3.7	6.19	6.22	1976.7984	1976.8133	989.4145	7.56
M5			6.19		1545.6080	1545.6159	773.8158	5.13
F(6)A1[3]G(4)1			6.22		1732.6925	1732.7027	867.3592	5.90
A2[6]G(4)1			6.26		1789.7139	1789.7243	895.8700	5.83
A2[3]G(4)1	14.93	0.1	6.38	6.38	1789.7139	1789.7277	895.8717	7.73
F(6)A2[6]G(4)1	15.50	26.2	6.53	6.57	1935.7719	1935.7879	968.9018	8.28
F(6)A2[3]G(4)1	15.90	8.9	6.69	6.70	1935.7719	1935.7909	968.9033	9.83
F(6)M5A1	16.19	0.1	6.89	6.79	1894.7453	1894.7575	948.3866	6.45
F(6)M4A1G(4)1	17.00	0.1	6.99	7.02	1894.7453	1894.7559	948.3858	5.61
F(6)A1G(4)1Ga(3)1			7.02					
M6			7.11		1707.6609	1707.6699	854.8428	5.24
M6D3			7.12					
Unknown 1	17.04	0.3		7.08		2023.7967	1012.9060	
M6D1	17.13	0.1	7.14	7.11	1707.6609	1707.6739	854.8448	7.58
A2G(4)1Ga(3)1			7.05		1951.7668	1951.7773	976.8965	5.40
A2G(4)2			7.10					
F(6)A3G(4)3S(3,3,3)3	17.59	0.2	7.41	7.26	2226.8673	2226.8785	1114.4471	5.04
F(6)A2[6]G(4)1Ga(3)1	18.11	5.5	7.38	7.44	2097.8247	2097.8311	1049.9234	2.93
F(6)A2G(4)2			7.43					
F(6)A2[3]G1Ga(3)1			7.55					
F(6)A2[3]G(4)1S(3)1			7.53		2226.8673	2226.8857	1114.4507	8.28
M4A1G(4)1Ga(3)1	18.44	0.3	7.42	7.56	1910.7402	1910.7535	956.3846	7.09
M5A1G(4)1			7.43					
F(6)M5A1G(4)1	18.84	0.1	7.74	7.70	2056.7981	2056.8093	1029.4125	5.51
F(6)M4A1G(4)1Ga(3)1	19.35	0.1	7.80	7.89	2056.7981	2056.8149	1029.4153	8.24
M7D3			7.81		1869.7137	1869.7299	935.8728	8.69
M7			8.0					
F(6)A2G(4)2S(3)1	19.96	0.4	8.12	8.11	2388.9201	2388.9299	1195.4728	4.16
F(6)A2G(4)2S(6)1	20.15	0.2	8.55	8.18	2388.9201	2388.9329	1195.4743	5.42
F(6)A2G(4)2Ga(3)1	20.51	0.1	8.25	8.32	2259.8775	2259.8989	1130.9573	9.27
F(6)M4A1G(4)1Sg(6)1			8.58		2201.8356	2201.8505	1101.9331	6.79
M8	21.8	0.1	8.84	8.85	2031.7665	2031.7779	1016.8968	5.88
M8D1D3			8.82					
M8D2D3			8.76					
F(6)A2G(4)2S(3,3)2	22.00	0.2	9.32	8.89	2680.0155	2680.0289	1341.0223	5.01

Nomenclature: F: fucose; G: galactose; Sg: *N*-glycolylneuraminic acid; Sx, number (x) of sialic acids linked to galactose; Ga: α1,3-linked galactose; A1: monoantennary; A2: biantennary; Mx: number (x) of mannose on core GlcNAcs; D1 indicates that the α1,2 mannose is on the Manα1,6Manα1,6 arm, D2 indicates that the α1,2 mannose is on the Manα1,3Manα1,6 arm, D3 indicates that the α1,2 mannose is on the Manα1,3 arm of M6 and on the Manα1,2Manα1,3 arm of M7 and M8; B: bisecting GlcNAc linked_1,4 to 1,3 mannose; Numbers with parentheses indicate the preceding monosaccharide’s linkage and those in brackets define to which core mannose is extended, if it needed to be defined. Numbers not in parentheses indicate the amount of the preceding feature. For example, F(6)A2[3]G(4)1Ga(3)1 represents a core fucosylated (α1,6-linked) bianntennary glycan with a β1,4-linked galactose directly attached to the α1,3-linked core mannose, and an α1,3-linked galactose attached to the β1,4-linked galactose.

**Table 4:** Number of structural elements found in USP mAb reference standards.

Structural Element	USP mAb 001	USP mAb 002	USP mAb 003
Total Glycans	47	45	40
High Mannose	12	12	10
Hybrid	11	10	11
Complex	23	22	18
Unknown *	1	1	1
Biantennary	22	25	26
Core Fucosylation	27	25	21
Sialylation	6	6	6
Alpha-Galactosylation	9	6	7

* Glycans observed that were not in the database library.

**Table 5:** Comparison of percent glycan abundance in USP mAb 001, mAb 002, and mAb 003 and reported values for NISTmAb. Glycans designated as “≤” co-eluted and may be present in a lower abundance than indicated. Glycans designated as “-” indicate that the F(6)A2[3]G(4)1 may have been included in the F(6)A2[6]G(4)1 results.

Glycan	USP mAb 001	USP mAb 002	USP mAb 003	NIST mAb *	NIST mAb **
F(6)A2	44.46	67.81	49.56	39.09	39
F(6)A2[6]G(4)1	27.17	16	26.2	28.12	37.8
F(6)A2[3]G(4)1	9.36	6.51	8.93	10.18	-
F(6)A2G(4)2	≤5.89	≤2.22	≤5.49	7.51	7.3
F(6)A1	1.98	1.71	1.99	2.127	2.5

* Results reported in Reference [1]; ** Result reported in Reference [3].

## Data Availability

Data sharing contains in this article.

## Disclaimer

Certain commercial equipment, instruments, vendors, or materials may be identified in this paper to specify adequately the experimental procedure. Such identification does not imply approval, endorsement, or certification by USP of a particular brand or product nor does it imply that the equipment, instrument, vendor, or material is necessarily the best available for the purpose or that any other brand or product was judged to be unsatisfactory or inadequate. All product names, logos, and brands are the property of their respective owners.

## Abbreviations

ACN: Acetonitrile; DMF, Dimethylformamide; ESI, Electrospray Ionization; FA, Formic Acid; FLR, Fluorescence; Gal, Galactose; gal-a1,3-gal, Two galactoses linked via an a1–3 bond; GlcNAc, N-acetylglucosamine; GU, Glucose Unit; GUH, b-N-acetylhexosaminidase; HILIC, Hydrophilic Interaction Liquid Chromatography; hr, Hour; IgG, Immunoglobulin G; kV, Kilovolt; M5, Mannose-5 glycan; mAb, Monoclonal antibody; min, Minute; mg, Milligram; mM, Millimolar; MS, Mass Spectrometry; ppm, Parts per million; QTOF, Quadrupole Time-of-Flight; RFMS, RapiFluor-MS; RM, Reference Material; SPE, Solid-phase extraction; mL, Microliter; UPLC, Ultra-Performance Liquid Chromatography; LC-MS, Liquid Chromatography Mass Spectrometry; V, Volt.
